# Early and Late Patient Outcomes in Urgent-Start Peritoneal Dialysis: A Prospective Study of Unplanned Initiation of Chronic Dialysis

**DOI:** 10.7759/cureus.31254

**Published:** 2022-11-08

**Authors:** Ghita El Bardai, Basmat Amal Chouhani, Nadia Kabbali, Adil Najdi, Mohamed Arrayhani, Tarik Sqalli Houssaini

**Affiliations:** 1 Department of Nephrology, Dialysis, and Transplantation, Hassan II University Hospital, Fez, MAR; 2 Laboratory of Epidemiology and Health Science Research (ERESS), Faculty of Medicine-Fez, Sidi-Mohammed-Ben-Abdellah University, Fez, MAR; 3 Epidemiology, Faculty of Medicine and Pharmacy of Tangier, Tangier, MAR; 4 Nephrology, Dialysis, and Transplantation, Souss Massa University Hospital, Agadir, MAR

**Keywords:** long-term outcome, short-term outcome, infectious complications, mechanical complications (mc), peritoneal dialysis (pd), urgent-start

## Abstract

Background: Peritoneal dialysis (PD) has become a well-established complementary alternative to hemodialysis (HD) as the first-line renal replacement modality. Unlike the temporary catheter for hemodialysis that can be used immediately after implementation, the PD catheter usage period remains controversial. The aim of this study was to compare the short- and long-term outcomes in patients under peritoneal dialysis according to the delay of starting the dialysis after catheter placement.

Methods: This observational prospective study was conducted over an eight-year and four-month period (from April 2014 to August 2021), including all patients treated with peritoneal dialysis for 18 months (from April 2014 to October 2015). The patients were divided into two groups according to whether the catheter was used during the first 15 days (PD-E) or 15 days after (PD-L) catheter placement. The primary outcomes were early complications (mechanical and infectious) within 90 days. Secondary outcomes included technique survival.

Results: Among the 36 patients included in the study, 14 started PD early (38.8%), while 22 started it 15 days after catheter placement (61.2%). The mean age between the two groups was not significantly different (41 ± 17 years vs 35 ± 16 years, p: not significant). There were no significant differences in the Charlson comorbidity index or the degree of autonomy. The incidence of infections was not significantly different between the two groups (13.6% in PD-L vs 21.4% in PD-E, p: not significant). The total number of mechanical complications was not significantly higher in the PD-E group compared to the PD-L group (42.8% vs 27.3%, respectively, p: not significant). Kaplan-Meier estimates of technique survival were comparable between the groups (log Rank: 1.908, p: 0.67).

Conclusions: Our study showed no increase in the risk of complications associated with early use of the PD catheter and no difference in technique survival. PD can be used as first-line renal replacement therapy in the unplanned initiation of chronic dialysis.

## Introduction

Peritoneal dialysis (PD) has long been an underutilized modality for renal replacement therapy (RRT) [1], despite other benefits, including preservation of residual renal function and a better quality of life [2]. Most patients who have started chronic dialysis are on hemodialysis (HD) by a central venous catheter (CVC) [3]. This increases the risk of complications such as infection, length of hospitalization, and mortality [3,4]. In April 2014, a rapid PD start-up program was set up at the Hassan II University Hospital, Fez, Morocco, the aim of which was to propose this technique as an initial modality of RRT in patients with chronic end-stage kidney disease (ESKD) in the event of an unplanned initiation of chronic dialysis. This program was started to expand access to care in our context, provide patients with the opportunity to choose the modality of RRT, and minimize the use of central venous catheters. Unlike the temporary catheter for HD that can be used immediately after implementation, the PD catheter usage period remains controversial. The latest guidelines from the International Society for Peritoneal Dialysis (ISPD) and European Best Practice Guidelines (EBPG) recommend waiting at least two weeks after peritoneal dialysis catheter insertion before commencing peritoneal dialysis [5, 6]. These recommendations are based on the fact that early catheter use increases the risk of mechanical and infectious complications. However, the evidence supporting these recommendations is weak. In addition, the long-term effects of early initiation of PD on patient outcomes remain unclear [7]. The aim of this study was to compare the short- and long-term outcomes in patients under PD according to the delay of starting the dialysis after catheter placement.

## Materials and methods

Study design

An observational and prospective study was carried out at the nephrology department of Hassan II University Hospital, Fez, Morocco. The study was conducted over an eight-year and four-month period from April 2014 to August 2021.

Patients

We included in the study all new patients with ESKD, defined as a creatinine clearance equal to or lower than 15 ml/min/1,73 m^2^ measured using the Modification of Diet in Renal Disease (MDRD) formula. These patients were treated with PD either immediately or on a planned basis. We divided patients into two groups according to the PD start time after placement of the catheter: Early-onset PD patients group (PD-E): beginning within the first 15 days of catheter placement, and patient group late-onset PD (PD-L): beginning at least 15 days after the catheter placement. We did not include patients presenting an absolute contraindication to PD.

Study progress

**Figure 1:**
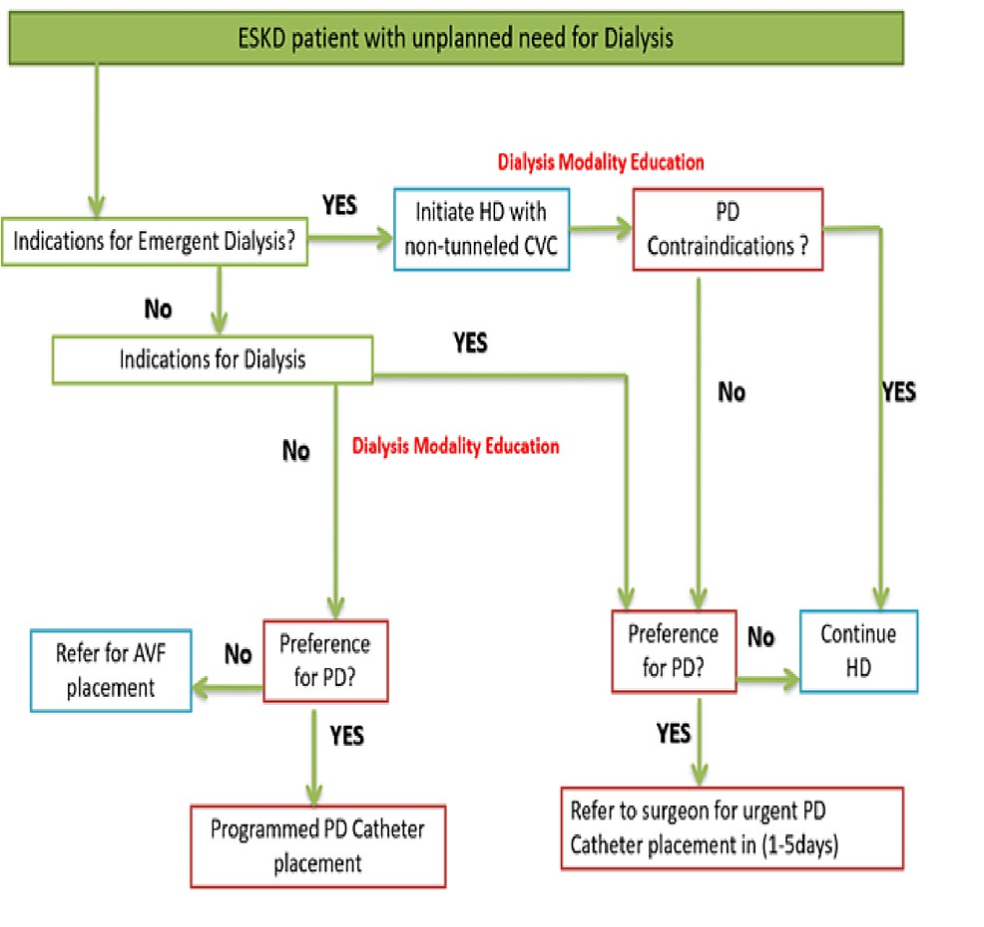
A flowchart describing steps involved in the evaluation and selection of late-presenting ESKD patients for urgent-start PD. ESKD: end-stage kidney disease; AVF: arteriovenous fistula; CVC: central venous catheter; HD: hemodialysis; PD: peritoneal dialysis

Late-referred ESKD patients were sorted into two groups: In patients without an emergency indication to start dialysis, the catheter placement for PD was done on a planned basis, and PD was started after an overall delay of two weeks. The second arm was composed of patients with an emergency need for dialysis (hyperkalemia, severe acidosis, severe volume overload, or marked uremia). For these patients, the PD catheter placement was performed with a delay of fewer than five days, preceded by one to three urgent HD sessions via temporary femoral vein access. PD was initiated with a break-in period of fewer than two weeks. All patients were offered an informed choice of dialysis modality. And any medical or social contradictions to PD were carefully checked (Figure 1).

Enrollment and follow-up time

The enrollment was conducted over an 18-month period from April 2014 to October 2015. All patients were followed until discharge from peritoneal dialysis. The follow-up was done by two nephrologists and a trained nurse.

PD prescription

**Figure 2:**
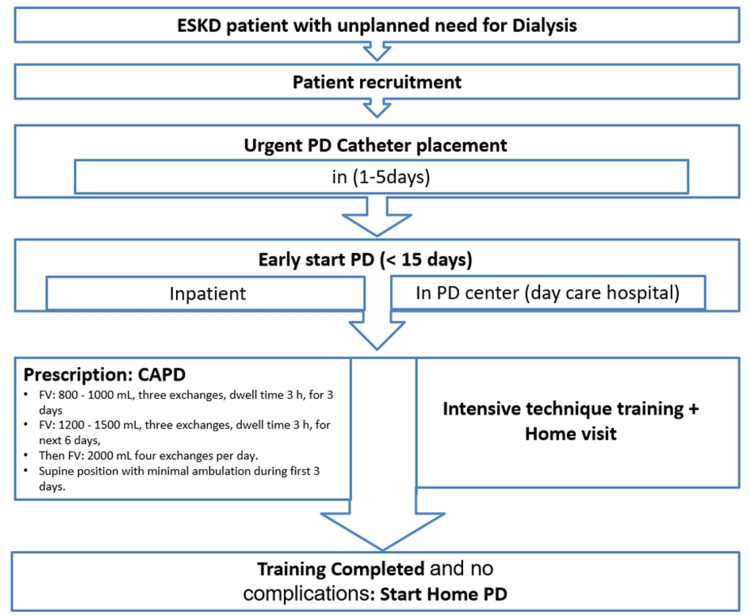
The flow chart of the urgent-start peritoneal dialysis program. FV: fill volume; PD: peritoneal dialysis; CAPD: continuous ambulatory peritoneal dialysis; ESKD: end-stage kidney disease

All patients were placed on continuous ambulatory PD (CAPD) for the first three months. In the PD-E group, daily exchanges of small volumes were made in parallel with intensive training of technique according to the following protocol in a supine position with minimal ambulation: for the first three days, three exchanges per day with a dwell time of three hours and a fill volume of 800 to 1000 ml for patients whose weight was <60 kg and ≥ 60 kg, respectively. During the next six days, there was an increase in fill volume from 1.2 L to 1.5 L for patients whose weight was <60 kg and ≥ 60 kg, respectively. Beyond this period, there was an increase in the number of exchanges to four exchanges per day and an increase in fill volume to 2 L-2.5 L depending on body surface area and intra-peritoneal pressure (Figure 2).

During the first two weeks in the PD-L group, the patients received technique training. After training was completed, patients started home PD with three to four exchanges per day, a dwell time of three hours, and a fill volume of 1500 ml to 1800 ml, which was progressively increased according to the body surface and the intraperitoneal pressure.

The tonicity of the dialysate was determined by the volume status (1.36% dextrose if there was no fluid overload, 2.27% or 3.86% dextrose if there was fluid overload). After three months, patients remained on CAPD or were converted to standard automated peritoneal dialysis.

The catheter placement

All of our patients have benefited from the following precautions before surgery: shower grooming with povidone-iodine or chlorhexidine soap, extensive shaving (nipples to mid-thigh), thorough cleaning of the umbilicus, locating the site of emergence and emptying the rectal ampulla.

The placement of the peritoneal catheter was performed in all our patients by a single surgeon, by laparoscopy, under general anesthesia. The catheter emergence site was located at the left lateral para-umbilical level in all cases. Intraoperatively, all our patients received prophylactic antibiotics with two grams of amoxicillin-clavulanic acid. The catheter used was the Tenckhoff type with a double sleeve, a swan neck, and a pigtail.

The PD nurses examined the catheter exit site and tunnel according to standard clinical practice. The first dressing of the catheter was done on the first day and then on the seventh day. Once the PD is started, the dressing was changed twice a week and or after each bath. A systematic peritoneal lavage was carried out with 500 ml of dextrose 1.36% on the first day to check the function of the catheter.

Data collection and clinical outcomes

We used patients' charts to complete the data collection forms. The data collected were: demographic data (sex, age, socio-economic status.), history (diabetes, hypertension.), causal nephropathy, comorbidity, and residual diuresis, Charlson comorbidity index (a comorbidity score validated for PD in 2001, considered as a major predictive factor for survival [8]), the delay between the catheter placement and the PD start, mechanical complications such as leaks (pericatheter, abdominal wall, and pleural), catheter blockage, and catheter migration, infectious complications (included exit-site infection, peritonitis, and tunnel infection. Complications were defined in accordance with the ISPD guidelines [6,9]), Status of patients at their latest news (switch to hemodialysis, transplantation, death), and cause of death.

The primary outcome measure was early complications (mechanical or infectious). Complications were examined at 90 days. Secondary outcome measures included technique survival rates and were evaluated after the first 90 days, two years, and at the end of the observation. These measures were taken from the time of commencement of PD exchanges and censored at the time of dialysis modality switch, transplantation, or death.

Statistical analysis

Data were entered on Microsoft Excel (Microsoft Corporation, Redmond, Washington, United States) and analyzed using IBM SPSS Statistics for Windows, Version 20.0 (Released 2011; IBM Corp., Armonk, New York, United States). A descriptive analysis was performed; quantitative variables were expressed as mean ± SD or median with interquartile range (IQR). Qualitative variables were expressed as percentages. The comparison of two means was carried out using the Student test, the comparison of two percentages was carried out by the Chi-squared test, and the comparison of two medians was performed by the Mann-Whitney test. Technique survival was estimated using the Kaplan-Meier analysis method; for comparing the technique survival, log rank test was used. The significance level used was p < 0.05.

## Results

Our study enrolled 36 patients; 20 males (55.6%) and 16 females. The mean age was 38.5 ± 16 years (range, 12-77 years). The percentage of diabetic mellitus patients was 11%. Hypertension was noted in 25% of patients. The mean Charlson comorbidity index was 2.61 ± 1.11. Half the patients were autonomous. The main background etiologies of ESKD were hypertension (14%), reflux and obstructive nephropathy (14%), diabetes mellitus (11%), and unknown (60%). The mean diuresis was 1032 ± 590 ml/day; oligo-anuria was noted in nine patients. The median break-in period (from catheter insertion to dialysis initiation) was 11.9 ± 6 days (0-22 days).

Fourteen patients started PD early (38.8%), while 22 patients started it 15 days after the catheter placement (61.2%). The comparison of patient demographics, clinical, and paraclinical data found there were no significant differences in terms of age, sex, Charlson comorbidity index, or in degree of autonomy between the two groups. Three diabetic patients were in the PD-E group, while only one diabetic patient was in the PD-L group (Table 1).

**Table 1:** The main demographic, clinical, and paraclinical characteristics of the study groups. PD-E group: early-start peritoneal dialysis group; PD-L group: late-start peritoneal dialysis group; eGFR: estimated glomerular filtration rate

Parameter	All patients (N=36)	PD-E group (N=15)	PD-L group (N =21)	p-value
Age (years) mean±SD	38.5±16.6	39.9±15.4	36.5± 18.5	0.36
Male n (%)	20(55.6%)	14(66.7%)	6(40%)	0.11
eGFR (ml/min /1.73m²) mean±SD	5.24±2.3	5.63±2.37	4.7±2.04	0.58
Diabetes mellitus n(%)	4(11.1%)	4(19%)	0(0%)	0.07
High blood pressure n(%)	9(25%)	7(33.3%)	2(13.3%)	0.17
Autonomy n(%)	32(88.9%)	18(85.7%)	14(93.3%)	0.47
Body mass index mean±SD	21.6±2.8	22±2.33	21.12±3.33	0.21
Charlson comorbidity index (CCI) mean±SD	2.75±1.1	3 ±1.18	2.4± 0.8	0.32
Serum albumin (g/dl) mean±SD	35.03±9.4	36.67± 11.3	32.73±5.43	0.46
Blood hemoglobin (g/dl) mean±SD	8.86±1.4	8.97±1.47	8.7±1.3	0.38
Calcemia mean±SD	89.9± 8.3	91.57±8.15	87.66±8.45	0.2

Both groups were closely followed, and the occurrence of complications was assessed at 90 days. We observed migration of the catheter in six patients, three of whom required surgical replacement. A peri-catheter leak was noted in four patients. It resolved spontaneously after a temporary stoppage of PD (an average time of 25.7 ± 26.1 days). Two cases of PD catheter obstruction were observed. The first was treated with urokinase. The second case was associated with peritonitis requiring catheter removal. Moreover, only one patient presented a parietal hematoma in the immediate postoperative period with a spontaneously favorable evolution. During the follow-up period, peritoneal fluid infection occurred in two patients. After adapted antibiotherapy, patients sterilized the peritoneal fluid, but one of them was transferred to HD because of the obstruction of the PD catheter, requiring its removal. Exit site infection was noted in four patients treated with antibiotics.

The incidence of complications was also compared. No statistically significant difference was found in the occurrence of complications between the two groups (Table 2). The incidence of infections was higher in the PD-E group, but the difference was not statistically significant (PD-L: 13.6% vs. PD-E: 21.4%, p>0.05). The total number of mechanical complications observed was not significantly higher in the PD-E group compared to the PD-L group (42.8% versus 27.3%; p>0.05).

**Table 2:** Peritoneal dialysis-related complications PD-E group: early-start peritoneal dialysis group; PD-L group: late-start peritoneal dialysis group

Complication	Overall (N=36)	PD-E (N=14)	PD-L (N=22)	p-value
Catheter migration	6	2	4	>0.05
Leakage	4	2	2	>0.05
Obstruction	2	2	0	>0.05
Exit site infection	4	2	2	>0.05
Peritonitis	2	1	1	>0.05

**Figure 3:**
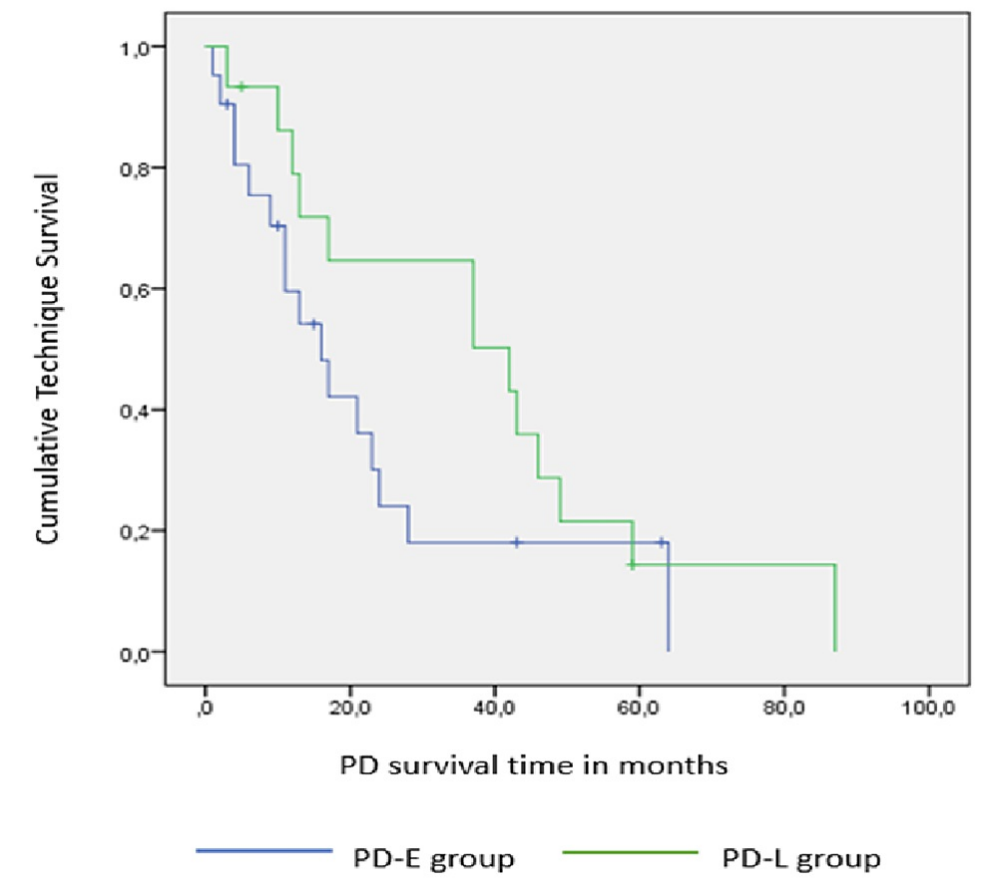
Technique survival curves for PD- E group and PD-L group. No significant difference between curves (log rank: 1.908, p: 0.67) PD: peritoneal dialysis; PD-E group: early-start peritoneal dialysis group, PD-L group: late-start peritoneal dialysis group

The median survival of the technique in all patients was 21 months IQR(11-46). Rates of technique survival for early- and late-start patients were 95% and 100% at 90 days, 36% and 65% at two years, and 19% and 17% at five years, respectively. A comparison of the two technique survival curves, according to the log-rank test, showed no significant difference in technique survival between the two groups, PD-E and PD-L (log rank: 1.908, p: 0.67) (Figure 3).

During the study period, three patients died of peritonitis (the three patients from the PD-E group). In two cases, the peritonitis was secondary to intestinal perforation; in the third case, the peritonitis was associated with the onset of septic shock after the operation. We noted four other deaths not related to the technique, one of which was secondary to severe acute respiratory syndrome coronavirus 2 (SARS-CoV-2). One patient was transplanted, and the other 28 patients were switched to HD, with the average switch time in months being 25.46 months ± 21.86 (2 - 92). The main causes of switching were patient choice in 39%, inadequate dialysis in 32%, catheter malfunction in 10.7%, and peritonitis in 17.8% of cases.

## Discussion

Except for a few countries and regions such as Mexico and Hong Kong, HD is still the dominant dialysis modality worldwide, with 70-80% of patients starting on HD [1]. In Morocco, many patients referred for dialysis do not have a distinct plan at the time of initiating dialysis therapy. In addition, some stable patients had acute worsening kidney function that was not predictable, resulting in an urgent need for dialysis. These patients are likely to be started on in-center HD with temporary vascular access known to be associated with excess mortality and increased risks of potentially lethal complications such as bacteremia and central venous thrombosis or stenosis [2,10,11]. Within the last decade, urgent-start PD has gained considerable interest among nephrologists. Several publications have provided assurances that urgent-start PD is indeed feasible and can serve patients well [7,10,12-14].

The EBPG state that the PD should be started two weeks after the implantation of the catheter [5]. Some even suggest waiting two to four weeks for better healing [15]. This dogma of starting PD after a delay of two weeks was challenged by several studies, which showed that the number of complications was not different between the patients who started PD early and those who started it later [2,7,10,12,13]. The risk of the urgent start of PD is the occurrence of mechanical complications, especially peritoneal fluid leakage. In our study, no significant difference was found between the two groups (14.2% PD-E vs. 9.09% PD-L, p: not significant). This complication rate was significantly higher in the urgent-start group, as reported by several other studies [16-18]. However, these results must be interpreted according to the catheter placement technique and the PD prescription. Thus, in a retrospective study including 657 patients with PD followed over a 10-year period, Liu et al. showed that abstinence from the use of the PD catheter for a certain period prior to the start of dialysis had no influence on the long-term survival of the catheter even though the total number of mechanical complications was higher in the early-PD group [19]. With regard to infectious complications, the majority of studies showed no difference between the urgent-start PD group and the planned-start PD group [16,18,19]. These data are confirmed by the results of our study.

It seems that after the initial phase of starting PD, complications tend to decrease in the long term. Results over 10 years of a retrospective study that evaluated 2059 urgent-start PD patients showed high rates of catheter patency and technique survival and low incidences of catheter-related complications [20]. Furthermore, an Australian randomized controlled trial concluded that urgent-start PD compared with conventional-start PD has acceptably low early complication rates and similar long-term technique survival [21].

In terms of mortality, Lobbedez et al. used data from the French Language Peritoneal Dialysis Registry to compare 568 patients with an urgent-start PD with 7931 who were on a programmed PD. No difference in mortality was observed between the two groups [22].

Comparing urgent-start PD to conventional-start PD data from a 2020 Cochrane review of nearly 3000 patients showed an increase in the risk of dialysate leak in patients receiving urgent-start PD. However, there were no demonstrated differences in the risk of catheter failures, infectious complications, technique, or patient survival [23].

In addition to this, a retrospective study compared the occurrence of complications in ESRD patients who initiated dialysis urgently according to whether they were treated in HD or in PD. The incidence of dialysis-related complications during the first 30 days was significantly higher in HD than in PD patients. HD patients had a significantly higher probability of bacteremia compared to PD patients. HD was an independent predictor of short-term dialysis-related complications. There was no significant difference between PD and HD patients with respect to the patient survival rate [24]. These findings were confirmed by a recent systematic review and meta-analysis [25].

## Conclusions

Our study showed no significant excess risk with the urgent start of PD in early and late outcomes. Clearly, PD can be used as first-line renal replacement therapy in the unplanned initiation of chronic dialysis. However, our study was based on a small sample of patients. Additional research and prospective randomized controlled trials are needed to determine the appropriate time to initiate PD after the insertion of a catheter.
